# Typing of *Candida* spp. from Colonized COVID-19 Patients Reveal Virulent Genetic Backgrounds and Clonal Dispersion

**DOI:** 10.3390/pathogens12101206

**Published:** 2023-09-29

**Authors:** Edith Quiroga-Vargas, Miguel Ángel Loyola-Cruz, Araceli Rojas-Bernabé, Mario Adán Moreno-Eutimio, Rodolfo Pastelin-Palacios, Clemente Cruz-Cruz, Emilio Mariano Durán-Manuel, Claudia Calzada-Mendoza, Graciela Castro-Escarpulli, Geovanni Hernández-Hernández, Mónica Alethia Cureño-Díaz, Verónica Fernández-Sánchez, Juan Manuel Bello-López

**Affiliations:** 1 Hospital Juárez de México, Mexico City 07760, Mexicomiguelqbp@gmail.com (M.Á.L.-C.); emilioduranmanuel@gmail.com (E.M.D.-M.); dracureno@yahoo.com.mx (M.A.C.-D.);; 2 Sección de Estudios de Posgrado e Investigación, Escuela Superior de Medicina, Instituto Politécnico Nacional, Mexico City 11340, Mexicocccalzadam@yahoo.com.mx (C.C.-M.); 3 Laboratorio de Investigación Clínica y Ambiental, Departamento de Microbiología, Escuela Nacional de Ciencias Biológicas, Instituto Politécnico Nacional, Mexico City 11340, Mexico; 4 Facultad de Química, Universidad Nacional Autónoma de México, Ciudad Universitaria, Mexico City 04510, Mexico; marioadan@quimica.unam.mx (M.A.M.-E.); rodolfop@unam.mx (R.P.-P.); 5 Facultad de Estudios Superiores Iztacala, UNAM, Tlalnepantla de Baz 54090, Mexico

**Keywords:** *Candida* spp., opportunistic infections, COVID-19, clonal dispersion, virulence

## Abstract

Advances in the knowledge of the pathogenesis of SARS-CoV-2 allowed the survival of COVID-19 patients in intensive care units. However, due to the clinical characteristics of severe patients, they resulted in the appearance of colonization events. Therefore, we speculate that strains of *Candida* spp. isolated from COVID-19 patients have virulent genetic and phenotypic backgrounds involved in clinical worsening of patients. The aim of this work was to virutype *Candida* spp. strains isolated from colonized COVID-19 patients, analyze their genomic diversity, and establish clonal dispersion in care areas. The virulent potential of *Candida* spp. strains isolated from colonized COVID-19 patients was determined through adhesion tests and the search for genes involved with adherence and invasion. Clonal association was done by analysis of intergenic spacer regions. Six species of *Candida* were involved as colonizing pathogens in COVID-19 patients. The genotype analysis revealed the presence of adherent and invasive backgrounds. The distribution of clones was identified in the COVID-19 care areas, where *C. albicans* was the predominant species. Evidence shows that *Candida* spp. have the necessary genetic tools to be able colonize the lungs, and could be a possible causal agent of coinfections in COVID-19 patients. The detection of dispersion of opportunistic pathogens can be unnoticed by classical epidemiology. Epidemiological surveillance against opportunistic fungal pathogens in COVID-19 patients is an immediate need, since the findings presented demonstrate the potential virulence of *Candida* spp.

## 1. Introduction

The immunological collapse of COVID-19 patients due to infection with the SARS-CoV-2 coronavirus generates high probabilities for the acquisition of healthcare-associated infections (HAIs) [1]. Due to the need for prolonged respiratory support in these patients, the most frequent HAIs are ventilator-associated pneumonias (VAPs) [2]. At the beginning of the pandemic, most of the literature had described the main causative agents of VAP in COVID-19 patients, among which antibiotic-resistant ESKAPE-group bacteria were recognized as one of the main problems in intensive care units (ICUs) [3,4,5]. Nevertheless, with the coming of the different pandemic waves due to new variants of SARS-CoV-2 and the prolonged stay of patients in the ICUs, new and diverse emerging and/or opportunistic pathogens were added, complicating the hospital stay of critically ill patients [6,7]. Even intestinal parasites, such as *Entamoeba* spp., *Hymenolepis nana*, *Schistosoma mansoni*, and *Trichuris trichiura*, have been linked to coinfection events with SARS-CoV-2. However, they were not associated with the complication of COVID-19 [8]. On the contrary, fungi (filamentous and yeasts) are pathogens that have been recognized as those that complicate the hospital stay of patients. These microorganisms have been reported as causative agents of colonizations, coinfections (superficial or invasive mycoses) in developed and developing countries. Examples of these opportunistic infections are aspergillosis caused by filamentous fungi of the genus *Aspergillus* (87.5%), mucormycosis caused by *Mucorales* spp. such as *Rhizopus* spp., *Mucor* spp., *R. arrhizus*, *R. microsporus*, *Lichtheimia* spp., and *Rhizomucor pusillus* (0.3 to 0.8%), and candidiasis caused by yeasts of the genus *Candida* (69%) [9,10,11,12,13,14]. In the case of colonization and acute lung infections by yeasts, *C. albicans* stands out as the most prevalent agent, followed by other species [15,16]. Other groups have reported *C. auris* as a causative agent of opportunistic infections and hospital outbreaks in COVID-19 patients, being a yeast of epidemiological importance worldwide [17,18,19]. The frequency of isolation of yeast causing coinfections and VAP-causing pathogens in COVID-19 patients depends, among other factors, on the adherence to good clinical practices by healthcare personnel in the management of COVID-19 patients, as poor handwashing and cleaning/disinfection of surfaces and medical equipment has been shown to directly impact on the clonal spread of nosocomial pathogens [5,20,21]. Other reasons besides ICU stay have been described as predisposing factors for the development of COVID-19-associated candidiasis, including prolonged use of antibiotics, corticosteroids, and iron and zinc deficiency [13]. Although *Candida* spp. have been detected in respiratory samples from humans with and without lung disease, their importance remains undetermined, as they have historically been considered a commensal organism with a low virulence potential. However, the dimorphic transition in vivo is considered indicative of an active infectious process [15,22].

Since some studies have shown yeasts of the genus *Candida* spp. to be responsible for causing coinfections in COVID-19 patients, it is necessary to know their role in terms of virulence. In previous studies by our working group, the microbiological diagnosis of VAP in COVID-19 patients led to the circumstantial identification of hidden outbreaks of *Pseudomonas aeruginosa* and *Acinetobacter baumannii*, so it is not unusual to speculate that the phenomenon of clonal dispersion by yeasts of the genus *Candida* spp. may also occur among colonized COVID-19 patients [5]. Therefore, defining the role of these pathogens through the genotyping of factors involved in yeast pathogenesis is of utmost importance, as this evidence would give greater importance to their detection in microbiology laboratories. In addition, determining the clonal dispersion of this type of microorganism in the ICU of COVID-19 patients highlights the need to innovate the epidemiological surveillance of HAIs and coinfections for decision-making in the management of critically ill patients. The aim of this work was the genotyping of *Candida* spp. related to colonization cases in severe COVID-19 patients through the search for genes involved in adhesion and invasion, as well as the analysis of genomic diversity through the analysis of intergenic spacer regions and their distribution in care areas for COVID-19 patients. In this work, we analyze and discuss the implications for the identification of *Candida* spp. clones with virulence potential that cause opportunistic infections in COVID-19 patients.

## 2. Materials and Methods

### 2.1. Ethical Considerations

The institutional Committee of Research, Ethics, and Biosafety of Hospital Juárez de México (HJM) approved the protocol under registration HJM 021/22-I in accordance with the Regulation of the General Health Law on Research for Health.

### 2.2. Population of COVID-19 Patients

A population consisting of 160 COVID-19 severe patients was included in the study between April and October 2020 (first wave of the pandemic). VAP in these patients was confirmed if patients met the inclusion criteria for suspected VAP (fever acquired after 48 h of intubation, purulent pulmonary secretions, leukocytosis, and infiltrates on chest X-ray) [23,24,25]. Empirical treatments for VAP were ceftriaxone and due to the national shortage of colistin, the final treatments were meropenem, imipenem, and piperacillin–tazobactam. Finally, a history of corticosteroid use was reported in all patients. For microbiological study, in aseptic conditions, COVID-19 patients were sampled through tracheal aspirates and the samples transported at 4 °C to the research laboratory for bacteriological culture (ESKAPE bacteria) by Loyola-Cruz et al., 2023 [5] and fungal culture (this work). Additionally, demographic data were obtained from medical records to describe the COVID-19 patient population (by age group and sex) and associated comorbidities.

### 2.3. Intentional Isolation of Yeasts from COVID-19 Patients

Tracheal aspirates were handled under a biosafety level 2 cabinet according to laboratory biosafety with the operator wearing a coverall protective gown. Prior to yeast isolations, direct observations under a microscope of the tracheal aspirate samples were made for the repeated observation of pseudomycelium or abundant presence of yeast non-pseudomycelium-forming. Those tracheal aspirates positive and abundant for the presence of pseudomycelium or abundant yeasts were cultured using calibrated microbiological loop (1 to 10 μL) on selective BiGGY agar (Becton Dickin-son & Co., Franklin Lakes, NJ, USA) and were incubated aerobically at 25 °C for 72 h. In this study, fungal cultures categorized as abundant were included when there were more than 10^5^ CFU/mL of tracheal aspirate (including pseudomycelium-forming and non-forming). Additionally, to rule out cases of contamination by fungal microbiota, a second sample was subjected to microbiological analysis (presence of pseudomycelium and abundant culture). Finally, for those cases with abundant double isolation of yeasts, colonial morphotypes and pigmentation were identified according to Nickerson et al., 1953 criteria [26]. Selected colonies were purified on PDA agar and grown in YPD broth, then frozen in glycerol (50%) and stored at −70 °C for future experiments.

### 2.4. Fungal Identification by MALDI-TOF Mass Spectrometry

Fungal isolates were identified by direct analysis of whole yeast cells using matrix-assisted laser desorption/ionization time-of-flight (MALDI-TOF) mass spectrometry by the Facultad de Química of Universidad Nacional Autónoma de México (UNAM). For this purpose, all strains were streaked in PDA agar and incubated at 25 °C for 48 h, and single colonies were subjected to identification using a Bruker MALDI Biotyper (Bruker Daltonik, Germany) according to the manufacturer’s instructions. The criteria for the best match in yeast protocol identification were scores above 2.0 (down to 3) for high-confidence identification.

### 2.5. Adherent Phenotype of Candida spp. Strains

Mature biofilms of *Candida* spp. were induced according to Stepanović et al. (2007) with minor modifications as follows [27]. *Candida* spp. strains were grouped by species and cultured in 3 mL of YPD broth with shaking at 25 °C for 48 h at 200 rpm. Cultures were centrifuged and a cold isotonic solution was added and adjusted to a 0.5 McFarland nephelometer. Cellular suspension was inoculated (10 μL) in YPD-broth (240 μL) into wells of a sterile 96-well polystyrene microtiter flat-bottom plate (per triplicate) (Thermo Electron Corporation, Corning, Corning, NY, USA). The plates were sealed and aerobically incubated at 25 °C for 72 h. Non-adherent cells were removed by aspiration and the wells were washed three times with sterile PBS 1X (pH 7.4) and stained with 250 μL of 0.1% crystal violet for 30 min at room temperature. The biofilms were subjected to solubilization with 250 μL of 30% (*v/v*) CH_3_COOH and quantified by measuring the corresponding OD_600_ using an Epoch^TM^, BioTek^TM^ spectrophotometer (Vermont). Inoculated wells with YPD media were used as negative controls. *Candida* spp. strains were classified based on their dye retention capacity as weak, moderate, and strong formers. The classification criteria were the OD values according to Stepanović et al. (2007) [27]. To gain insight into the possible chemical nature of the molecules involved in adhesion, the biofilm formation assay was repeated with pretreatment with proteinase K (1 mg/mL in NaCl 100 mM/Tris 200 mM buffer, pH 7.5) at 56 °C for 2 h [28,29]. Differences between the biomass of biofilms with and without proteinase K treatment were determined, and a chi-squared test (*p* = 0.05) was used to determine differences between the two assays.

### 2.6. DNA Extraction of Candida spp. Strains

For genotyping assays of *Candida* spp. strains, genomic DNA was isolated using the Favorgen^®^ Genomic DNA Kit Commercial (Omega BIO-TEC, GA, SA) with minor modifications as follows. Fungi cells were subjected to pretreatment of 30 min at 30 °C in the presence of lyticase (Sigma-Aldrich^®^, St Louis, MO, USA) and sorbitol buffer, followed by a final treatment with proteinase K at 60 °C overnight. Integrity of genomic DNA was visualized on horizontal 0.8% agarose gels, and was used as template in PCR assays as follows.

### 2.7. Detection of Virulence Genes of Adherence and Invasion in Candida spp. Strains

The virulence genes *ALS1* (agglutinin-like sequence type 1), *HWP1* (integrin-like protein alpha), *INT1* (hyphal wall protein 1) and *SAP1* (secreted aspartyl proteinase 1) were detected by endpoint PCR [30,31,32]. Amplicons were visualized on horizontal agarose gels and staining with ethidium bromide. With the information obtained, a genotyping analysis of the genes involved in cell adhesion and invasion (*ALS1*/*HWP1* versus *INT1*/*SAP1*) was performed. Primers used for this purpose are shown in Table 1.

### 2.8. Search for Clones through Molecular Typing of Candida spp. by T3B Method

*Candida* spp. strains were subjected to molecular typing by the T3B method using the universal primer T3B previously described by Thanos et al. (1996) [33] with minor modifications, as follows (Table 1). The total reaction volume was 50 μL and consisted of molecular biology-grade water, 1× PCR buffer, 20 nM MgCl_2_, 25 mM deoxyribonucleotide phosphate, 100 pM of T3B primer, *Taq* DNA polymerase (1U) (Thermo Scientific, Foster City, CA, USA) and 300 ng of template DNA. Amplification conditions were pre-denaturation at 95 °C for 5 min, denaturation at 95 °C for 30 s, annealing at 35 °C for 1 min, and extension at 72 °C for 1.5 min, with a final extension at 72 °C for 5 min at the end for 34 cycles. Genetic profiles were run in 1× TBE buffer, pH 8.3, and separated in horizontal electrophoresis in 1.5% agarose gels, visualized, photographed under UV illumination, and analyzed by intra-gel pattern comparison using ImageLab 5.2.1. To confirm the reproducibility of T3B, PCR assays were performed three times. Tenover criteria was used to establish the clonal relationship between yeasts with the same genus and specie [34]. The distance matrix was calculated using a linear semilogarithmic method (ImageLab 5.2.1 and Microsoft Excel 16.76). The dendrograms were constructed using the UPGMA algorithm, with the Dice similarity index and a bootstrap of 1000 repetitions using the Past4 program (Version 4.09, Oslo, Norway).

## 3. Results

### 3.1. Description of Population of COVID-19 Patients

During the period from April to October 2020 (first wave of the pandemic), a cohort of patients COVID-19 were admitted to HJM (*n =* 160). According to the CO-RADS classification system, these patients were classified to a degree of severity as reported by Prokop et al. (2020) [35]. The study population was classified as critical and were subjected to mechanical endotracheal intubation. COVID-19 patients were cared for in seven designated COVID-19 areas (A, B, C, D, E, F, and G) previously described by our group [5]. Demographic information of this population revealed that 31.9% (*n* = 51) and 68.1% (*n* = 109) belonged to the female and male sex, respectively. Table 2 shows the demographic distribution of COVID-19 patients by sex and age group admitted in the ICU of HJM. The predominant comorbidities in the study population were obesity, diabetes, and hypertension. Interestingly, these comorbidities were mainly present in the population between 36 and 64 years of age and who received corticosteroid therapy during the COVID-19 illness (Table 2). In this COVID-19 patient population, ESKAPE and non-ESKAPE bacteria were detected in tracheal aspirates in 93.8% (*n* = 160), according to Loyola-Cruz et al. (2023) and Durán-Manuel et al. (2022) [5,20].

### 3.2. Candida spp. Isolation from Colonized COVID-19 Patients

**Figure 1:**
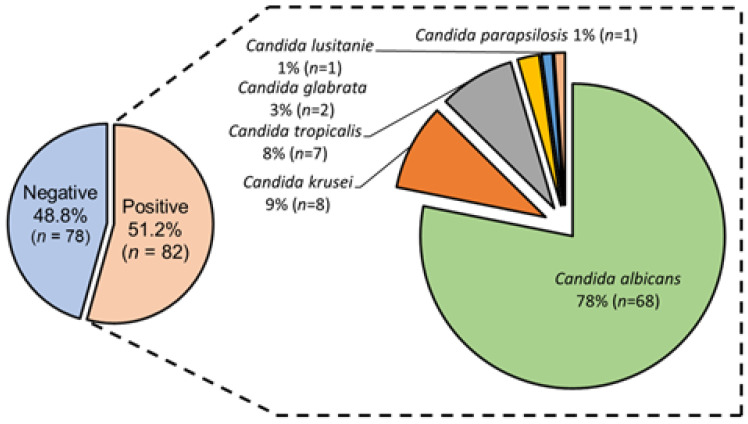
Taxonomic distribution of isolates of *Candida* spp. by species isolated from colonized COVID-19 patients admitted in the ICU of Hospital Juárez de México.

Mycological analysis by classical microbiology revealed that 51.2% (*n* = 82) of the tracheal aspirates of COVID-19 patients showed fungal growth. Therefore, these patients were classified as colonized by *Candida* spp. The absence of mycological growth in the remaining patients 48.8% (*n* = 78) was confirmed through a second culture. Polymicrobial by fungi (two yeast morphotypes) and monomicrobial (one yeast morphotype) cultures were identified in 5.7% (*n* = 5) and 94.3% (*n* = 82), respectively. Colonized patients classified in the adult age group (36–64 years old) were those who developed polymicrobial infection by two fungi. Infections by only one yeast were heterogeneously identified in all four age groups. Through MALDI-TOF technology, fungi isolates were taxonomically classified in six species of *Candida* spp. Proteomic profiles of *Candida* spp. strains, showed that *C. albicans* was the predominant species, followed by *C. krusei*, *C. tropicalis*, *C. glabrata*, *C. parapsilosis*, and *C. lusitanie.* One genus and one species of fungi implicated in colonization in a COVID-19 female adult patient were identified (*Wickerhamomyces anomalus*). No preferential distribution by *Candida* spp. species or age group of patients was identified. Figure 1 shows the taxonomic distribution of isolates of *Candida* spp. by species isolated from colonized COVID-19 patients.

### 3.3. Candida spp. Strains Showed High Adherence Capacity on Polystyrene

To determine the possible virulent potential of *Candida* spp. strains isolated from colonized COVID-19 patients, the adherent phenotype of the isolates on polystyrene was analyzed. The results showed that 52.9% of the isolates were classified as strongly biofilm-producing, where *C. albicans* and *C. krusei* were the predominant species in this classification. Only a few isolates of *C. glabrata*, *C. lusitanie*, and *C. parapsilosis* species were classified as strong producers. Only four isolates (4.6%) of the total population did not show the adherent phenotype. Table 3 shows the classification of *Candida* spp. isolates according to their biofilm-forming ability by biomass quantification at 600 nm (non-forming, weak, moderate, and strong).

### 3.4. Disruption of Mature Biofilms of Candida spp. Show a Possible Virulent Genotype

**Figure 2:**
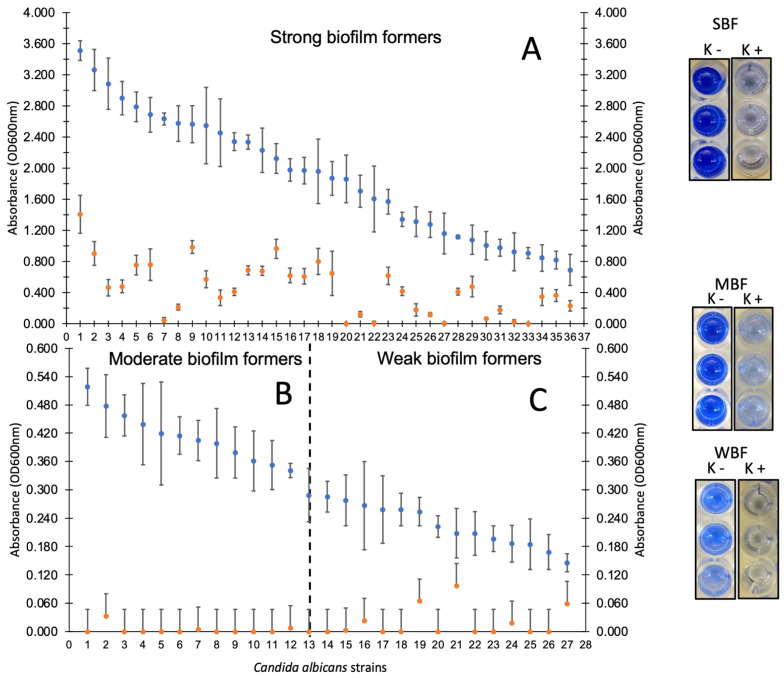
Adherent phenotype of the population of *C. albicans* strains isolated from colonized COVID-19 patients on polystyrene. (•) Without treatment with proteinase K. (•) With treatment with proteinase K (1 mg/mL). (**A**) Strongly producing strains, (**B**) moderately producing strains, and (**C**) weak biofilm-producing strains.

The strains of *Candida* spp. that were categorized as strong, moderate, and weak biofilm producers were subjected to unspecific proteolytic treatment with proteinase K. This was done with the aim of finding out the possible chemical nature of the molecules involved in polystyrene adhesion. The results revealed that for all species (regardless of their adherent classification), the disruption associated with proteinase K treatment was significant (*p* = 0.05). Figure 2 shows the adherent phenotype results for the population of *C. albicans* strains (with and without proteinase K treatment) with strong, moderate, and weak biofilm-forming characteristics where a significant reduction in biomass quenching after proteolytic treatment is observed. The disruption of mature biofilms of *C. krusei*, *C. tropicalis*, *C. glabrata*, *C. lusitanie* and *C. parapsilosis* after treatment with proteinase K is shown in the Supplementary Materials.

### 3.5. High Frequency of Candida spp. with Virulent Genetic Background

To provide clinical value to *Candida* spp. strains and to correlate their adherent phenotypes, a search for four genes coding for virulence factors in this fungus, cell adhesion and invasion was carried out. The analysis of the relationship between virulence genes revealed that 100% of the *C. albicans* isolates have the necessary genotype to be able to adhere and invade the host cell (*ALS1^+^/INT1^+^/SAP1^+^* and *HWP1^+^/INT1^+^/SAP1^+^*). In the case of *C. krusei* and *C. tropicalis* isolates (second and third prevalent species), they showed the following adherence/invasion genotypes: *ALS1^+^/INT1^+^*, *HWP1^+^/INT1^+^*, *ALS1^+^/SAP1*^+^ and *HWP1^+^/SAP1^+^*. For the remaining two species, the detected genotypes were only associated with adhesion genes *ALS1^+^* and *HWP1^+^*, with no association with invasion genes. Table 4 shows the frequencies of genotypes detected by PCR in *Candida* spp. (*ALS1*, *HWP1*, *INT1*, and *SAP1*).

### 3.6. Molecular Typing of Candida spp. by T3B Method

Due to reduced diversity of adherence and invasion genotypes identified in the *Candida* spp. isolates, we speculated on possible clone dispersion among COVID-19 patients. To clarify this hypothesis, molecular typing of *Candida* spp. isolates (grouped by species) for genomic diversity was performed by PCR amplification of intergenic spacer regions by the T3B primer method. *Candida parapsilosis* (*n =* 1) and *C. lusitanie* (*n =* 1) were not included in this test. Profiles of the intergenic spacer regions revealed sizes of amplicons from *Candida* spp. ranged from slightly more than ≈400 bp to about ≈4500 bp. Additionally, the analysis of the similarity (by dendrogram construction) of the isolates showed that the genomic diversity of *C. albicans* showed that the 68 isolates were grouped into three clonal clusters (CaG1, CaG2, and CaG3), consisting of 64, three, and one strain, respectively. Therefore, all isolates of *C. albicans* were grouped in three unique strains.

**Figure 3:**
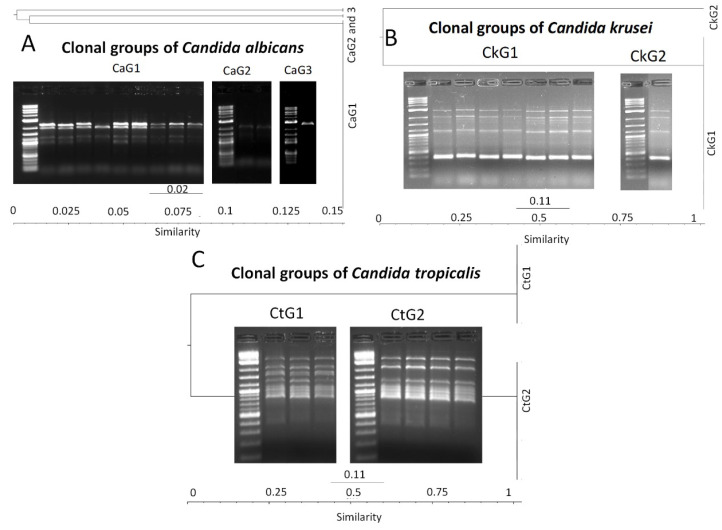
Similarity analysis of genomic diversity (by dendrogram construction) and representative profiles of the intergenic spacers by T3B method of *Candida* spp. isolated from colonized COVID-19 patients. (**A**) Clonal groups of *C. albicans*. M. Molecular weight marker (2 log), lines 1–9: *C. albicans* strains. (**B**) Clonal groups of *C. krusei*. M. Molecular weight marker (2 log), lines 13–20: *C. krusei* strains. (**C**) Clonal groups of *C. tropicalis*. M. Molecular weight marker (2 log), lines 21–24: *C. tropicalis* strains. Note: The gel images were cropped to show only representative profiles..

Moreover, *C. krusei*, *C. tropicalis*, and *C. glabrata* isolates were grouped in two clonal groups (CkG1/7 and CkG2/1), one clonal group (CtG1/3 and CtG/4) and one clonal group (CgG1/2), respectively. The genomic diversity of *Candida* spp. clones isolated from colonized COVID-19 patients was shown through the construction of dendrograms (Figure 3).

### 3.7. Candida spp. Clonal Dispersion Investigation in COVID-19 Areas

**Figure 4:**
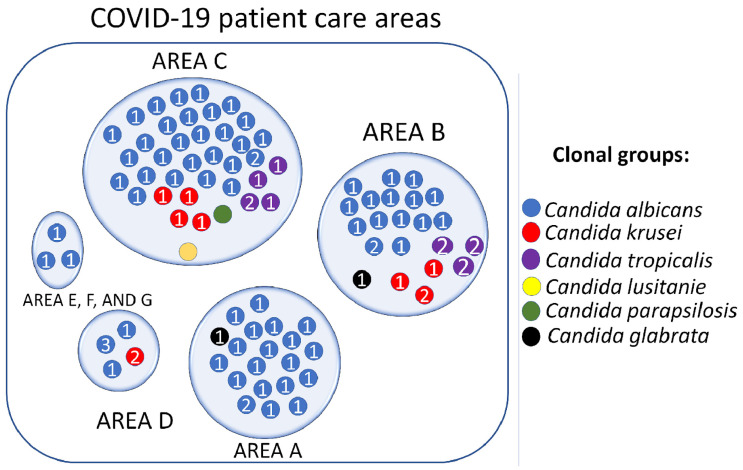
Spatial distribution of *Candida* spp. clones isolated from colonized COVID-19 patients by care areas of Hospital Juárez de México.

The analysis of the dispersion of *Candida* spp. clones among colonized COVID-19 patients was performed according to the classification by patient care areas as previously reported by Loyola-Cruz et al., 2023 [5]. Clonal groups of the predominant species (CaG1, CaG2, and CaG3) were identified in the seven care areas. COVID-19 B and C showed the highest diversity of clonal groups of all identified *Candida* spp. Species with low frequency were also involved in dispersal events in the other COVID-19 patient care areas. Figure 4 shows the clonal distribution of clonal groups of *Candida* spp. by COVID-19 patient care areas (A, B, C, D, E, F, and G).

## 4. Discussion

Among the adverse events that can occur in critically ill patients, including those with COVID-19, are the development of HAIs, of which VAP is one of the most important. HAIs play a crucial role in patient morbidity and mortality, since it has been shown that due to the absence of specific therapies to combat the new SARS-CoV-2 coronavirus infection, alternative therapies were employed that were initially promising, but over the course of the pandemic had serious consequences [16,36,37]. The use of corticosteroids, empirical antibacterial and antifungal therapy, the presence of comorbidities, and mechanical intubation itself, among others, have been recognized as potential factors that may lead to the development of colonization and subsequent coinfections associated with mechanical ventilation, among which fungi are recognized as one of the main causative agents of both superficial and systemic opportunistic infections, complicating the condition of COVID-19 patients [38,39]. The epidemiology of this type of infection indicates that regardless of the degree of development of the countries, severe COVID-19 patients have acquired various mycoses (superficial and systemic) with high frequencies. For example, for *Aspergillus* spp., this has been 87.5%, mucormycosis 0.3–0.8% and candidiasis 69% [9,10,11,12,13,14]. Under this context, in the present study, we demonstrated that several species of the genus *Candida* were related to colonizing in COVID-19 patients treated at the HJM. As can be seen in Table 1, the adult male patient population (36–64 years old) showed the highest incidence of colonization by *Candida* spp. An analysis of the scientific literature on coinfections in COVID-19 patients from 23 countries showed that the male sex (72.9%) was the most susceptible to acquire a fungal coinfection, in contrast to a 25.9% incidence in the female sex [40]. This work mentions that sex hormones and X chromosomes involved in innate and adaptive immunity may play an important role in susceptibility to COVID-19 infection in men. Consequently, sex would be considered a risk factor for greater morbidity and severity in male patients with COVID-19.

Furthermore, analysis of the distribution of *Candida* spp. identified showed that *C. albicans* was the predominant species in our study population (Figure 1). In several countries around the world, *C. albicans* has been reported as the most important species in mycotic infections of COVID-19 patients [15,41,42]. The other less frequently identified *Candida* spp. have also been reported in these patients [43,44,45]. Interestingly, the second-most prevalent species was *C. krusei* (9%), since in contrast to other studies, species such as *C. glabrata* and *C. tropicalis* have been identified as the second-most infectious agents in COVID-19 patients [46]. In recent years, a significant increase in the incidence of this species in pediatric patients has been identified (from 4.93% to 44.09%) [47]. We speculate that various factors, such as the common resistance to fluconazole in this species, as well as the possible emergence of virulent strains, directly impact isolation rates, and can be considered potential emerging pathogens. According to the scientific literature, the recommended antifungal treatment for this type of infection in COVID-19 patients has been amphotericin B (50%) and voriconazole (22.16%) [40]. Nevertheless, the low frequency of isolation of these *Candida* spp. has led to uncertainty about their clinical value, so it was necessary to analyze the phenotype and genotype of these species together with the predominant species of *C. albicans* to establish the basis for their virulence and resistance. Moreover, the presence of *C. auris* has been reported in Mexico and other countries in the world. This is one of the species that has attracted attention in the COVID-19 pandemic due to their epidemiological importance, due to the appearance of isolates resistant to multiple antimycotics and which has also been the cause of outbreaks among COVID-19 patients [18,48,49]. Since *C. auris* is a global threat due to its high transmission in hospitals and healthcare facilities, the intentional search for this species is necessary to detect epidemiologically important lineages, such as those already identified in the USA, where the presence of African and Asian lineages was revealed in COVID-19 patients [50]. Finally, it is important to mention that among the weaknesses of this study is the technology used to identify fungal isolates. This is due to the fact that there are reports of coincidence levels in identification by MALDI-TOF mass spectrometry greater than 93%. In contrast, other works report 99% and 100% coincidence between a mass spectrometry method versus sequencing of the ITS region [51,52].

To provide information about the virulence potential of *Candida* spp., we address one of the main characteristics of fungi to initiate an infectious process—adherence. It is recognized that adherence of microorganisms to host tissues is the prerequisite for the initiation of their pathogenesis. Numerous studies have shown that fungal adhesins are the molecules involved in cell adhesion [53,54,55]. Some of these biomolecules have been extensively characterized and have also been shown to be involved in morphological changes in the fungus [56,57]. Several adhesins in *Candida* spp. have been studied, such as Ala1p, Als1p and Hwp1p, Csh1, Ywp1, Pra1 and Saps, which are necessary for generating infectious processes [58,59,60]. The adherent ability of the various *Candida* spp. was analyzed by quantifying the biomass generated on polystyrene plates.

The identification of *C. albicans*, *C. tropicalis*, *C. glabrata*, *C. lusitanie*, and *C. parapsilosis* isolates as strong biofilm producers and the detection of their disruption upon proteinase K treatment suggested the presence of a virulent genetic background associated with the presence of adhesins of a proteinaceous nature (Figure 2 and Table 3). To address this hypothesis, we searched for genes associated with adhesion (*ALS1/HWP1*) followed by genes involved in cell invasion (*INT1/SAP1*). The analysis of genotypes in the *Candida* spp. population showed two important findings: the presence of genotypes that could confirm the adherent phenotype, followed by the possible ability to invade the host through cell proteolysis (Table 4). The adhesins ALS1 and HWP1 have been reported to be most prevalent in *Candida* spp. isolated from various infectious processes [60,61], so it is not difficult to speculate that these two adhesins are widely distributed in the *Candida* spp. genus regardless of the infectious focus, in this case lung infections in COVID-19 patients. The presence of the *INT1* and *SAP1* genes are considered necessary to induce the morphogenesis of the fungus to a yeast-like state and the expression of proteases necessary for cell invasion. These two genes are recognized as potential virulence factors that fungi employ after adherence [62,63,64]. Future works are aimed at analyzing the expression of these genes by in vitro infection processes on A549 cell cultures of basal alveolar epithelium to confirm the involvement of these four genes during fungal pathogenesis. The homogeneity in the genotypes identified allowed us to speculate on the possible genomic relationship between the different *Candida* spp., which could be interpreted as clonal dispersion of *Candida* spp. in COVID-19 patient care areas.

To confirm this hypothesis, molecular typing of the isolates was performed by PCR assays using the T3B primer method. The results of similarity comparison (by dendrogram construction) between intergenic spacers obtained by PCR assays showed a close genomic relationship between the populations of *C. albicans*, *C. krusei* and *C. tropicalis* species (Figure 3). The latter confirmed the dispersion of clonal groups CaG1, CaG2, CaG3 for *C. albicans*, CkG1 and CkG2 for *C. krusei*, and CtG1 and CtG2 for *C. tropicalis.* The T3B typing method has been widely used for genomic diversity in *Candida* spp. [65,66] and has been suggested as a potential tool for clone detection due to its high specificity and reproducibility. Other alternative methods for clone detection have been reported, such as sequencing of the *ERG11* gene involved in antifungal resistance. Corzo-Leon et al. (2021) characterized and confirmed a hospital outbreak of *C. parapsilosis* by identifying mutations in the *ERG11* gene [67]. Future works will focus on the genetic variability of the *ERG11* gene and its relationship with antifungal resistance in the isolates of this study. It is important to mention that due to the emergence of emerging and reemerging pathogenic microorganisms, it is necessary to use molecular epidemiology as a tool to analyze their dispersion. Although the *Candida* species identified in the present work were not of the *C. auris* species, their virulent genetic background was the reason for this study on their dispersion.

Conversely, it will also allow us to confirm the findings obtained in this work on clonal dispersion through the T3B fingerprint method. The information generated by T3B molecular typing allowed the construction of a clone distribution map in the seven COVID-19 patient care areas of the HJM (Figure 4). In general, a wide distribution of *Candida* spp. clones was identified in the COVID-19 care areas, where *C. albicans* was the species that—in addition to being the predominant species—was the one with the highest clonal dispersion.

The distribution of the CaG1 clonal group was identified in all seven focus areas, followed by the CkG1 clonal group that was identified in focus areas B, C, and G. The CtG1 and CtG2 clonal groups were concentrated in the B and C areas. Factors that influence the spread of microbial clones in ICUs and lead to outbreaks have been recognized. In our working group, we have demonstrated by molecular techniques the spread of antibiotic-resistant *A. baumannii* and *P. aeruginosa* clones in patients and medical equipment used with COVID-19 patients. In these works, we documented areas for improvement for a reduction in VAP incidence, such as adherence to good clinical practices, handwashing by healthcare personnel, implementation of cleaning procedures, and high-level disinfection [5,20,68]. The identification of *Candida* spp. species with identical genetic backgrounds confirms the role of healthcare workers in the emergence of HAIs in critical areas through the unintentional spread of pathogens. Without a doubt, understanding fungal infection in critically ill patients is a challenge, since although the clinical characteristics, symptoms and laboratory diagnosis (including biopsy analysis to confirm fungal infection) are of great value, it is necessary to know the role of these as causal agents that could displace the pulmonary microbiota as the case of the ESKAPE bacteria [69]. Because the lung fungal load is usually low in healthy individuals, under conditions of immunosuppression due to comorbidities or use of corticosteroids, some communities can colonize and cause infection [41,70]. Currently, research on the lung mycobiome is limited, so it is necessary to generate scientific evidence demonstrating that the fungal microbiome is altered in patients with COVID-19. An association between SARS-CoV-2 infection and pulmonary dysbiosis has been reported, due to changes towards colonization by *Candida* spp. and a decrease in fungal diversity [41]. The information generated through these studies can be used to understand the dynamics of these populations during their pathogenesis and explore antifungal treatments in critically ill COVID-19 patients.

## 5. Conclusions

This work shows the importance of epidemiological surveillance against other types of microorganisms different from the ESKAPE group, since the complexity of COVID-19 patients generates susceptibility to an infinity of infections, where not only bacteria of the ESKAPE group may be involved as causative agents of HAIs, colonization and opportunistic infections. Nevertheless, continuing with strategies such as the limited use of corticosteroids, adherence to handwashing by health personnel, isolation of confirmed cases of fungal infections, and the use of high-level disinfection methods continue to be the best strategies for the management of critically ill patients, with the aim of reducing or avoiding the appearance of opportunistic infections. Similarly, the findings presented demonstrate the need for the implementation of molecular methods that allow us to understand the chain of transmission of pathogens to detect hidden outbreaks by opportunistic pathogens and reveal associations that are not visible to classical epidemiology.

## Figures and Tables

**Table 1:** Primers used in this study.

Gene	Target	Sequence (5′→3′)	Size Amplicon (bp)	Reference
*ALS1*	Agglutinin-like sequence type 1	ALS1F: GAC TAG TGA ACC AAC AAA TAC CAG A	318	[30]
		ALS1R: CCA GAA GAA ACA GCA GGT GA		
*INT1*	Integrin-like protein alpha	INT1FP: AAG CTC TGA TAC CTA CAC TAG CGA	239	[31]
		INT1RP: GTT AGG TCT AAA GTC GAA GTC ATC		
*HWP1*	Hyphal wall protein 1	HWP1F: ATG ACT CCA GCT GGT TC	572	[32]
		HWP1R: TAG ATC AAGAAT GCA GC		
*SAP1*	Secreted aspartyl protease 1	SAP1F: GCT CTT GCT ATT GCT TTA TTA	253	[30]
		SAP1R: CAT CAG GAA CCC ATA AAT CAG		
*T3B*	Intergenic spacer regions	AGG TCG CGG GTT CGA ATC C	Variable	[33]

**Table 2:** Demographic distribution of COVID-19 patients categorized by sex and age group admitted in the ICU of *Hospital Juárez de México*.

Patient	Gender		Corticosteroid	Associated Comorbidities, *n* (%)				Other
Classification	*n* (%)		Therapy					*n* (%)
			*n* (%)	Diabetes	Hypertension	Renal	Obesity	Cancer
	Male	Female				Insufficiency		
Pediatric ^a^	2 (1.8)	5 (9.8)	0 (0)	0 (0)	0 (0)	0 (0)	5 (3.6)	3 (100)
Young adults ^b^	10 (9.2)	6 (11.8)	8 (5.8)	5 (3.9)	7 (6.1)	0 (0)	15 (10.7)	0 (0)
Adults ^c^	79 (72.5)	29 (56.9)	102 (73.4)	96 (75)	85 (74.6)	12 (85.7)	97 (69.3)	0 (0)
Elderly ^d^	18 (16.5)	11 (21.6)	29 (20.8)	27 (21.1)	22 (19.3)	2 (14.3)	23 (16.4)	0 (0)
Total	109 (100)	51 (100)	139 (100)	128 (100)	114 (100)	14 (100)	140 (100)	3 (100)

^a^ 0–18 years old, ^b^ 19–35 years old, ^c^ 36–64 years old, ^d^ >65 years old.

**Table 3:** Classification of *Candida* spp. strains isolated from colonized COVID-19 patients from Hospital Juárez de México according to their biofilm formation.

Species of *Candida*	Classification of Biofilm Forming, *n* (%)			
	Non-Forming	Weak	Moderate	Strong
*C. albicans* (*n* = 68)	3 (4.4)	18 (26.5)	11 (16.2)	36 (52.9)
*C. krusei* (*n* = 8)	0 (0)	4 (50)	4 (50)	0 (0)
*C. tropicalis* (*n* = 7)	0 (0)	0 (0)	0 (0)	7 (100)
*C. glabrata* (*n* = 2)	1 (50)	0 (0)	0 (0)	1 (50)
*C. lusitanie* (*n* = 1)	0 (0)	0 (0)	0 (0)	1 (100)
*C. parapsilosis* (*n* = 1)	0 (0)	0 (0)	0 (0)	1 (100)
Total (*n* = 87)	4 (4.6)	22 (25.3)	15 (17.2)	46 (52.9)

**Table 4:** Frequencies of genotypes of adherence (*ALS1*, *HWP1*) and invasion (*INT1*, *SAP1*) detected by endpoint PCR in *Candida* spp. isolated from colonized COVID-19 patients admitted in the ICU of Hospital Juárez de México.

**Adherent genotype, *n* (%)**		***Candida* spp.**	**Invasive Genotype, *n* (%)**	
			***INT1***	***SAP1***
	***ALS1***	***C. albicans* (*n* = 68)**	68 (100)	68 (100)
	***HWP1***		68 (100)	68 (100)
	***ALS1***	***C. krusei* (*n* = 8)**	6 (75)	0 (0)
	***HWP1***		4 (50)	0 (0)
	***ALS1***	***C. tropicalis* (*n* = 7)**	1 (14.2)	1 (14.2)
	***HWP1***		1 (14.2)	1 (14.2)
	***ALS1***	***C. glabrata* (*n* = 2)**	2 (100)	0 (0)
	***HWP1***		0 (0)	0 (0)
	***ALS1***	***C. lusitanie* (*n* = 1)**	0 (0)	0 (0)
	***HWP1***		0 (0)	0 (0)
	***ALS1***	***C. parapsilosis* (*n* = 1)**	0 (0)	0 (0)
	***HWP1***		1 (100)	0 (0)

## Data Availability

Not applicable.

## Supplementary Materials

The following are available online at https://www.mdpi.com/article/10.3390/pathogens12101206/s1, Figure S1: Adherent phenotype of the population of *C. krusei* (D), *C. tropicalis* (E), *C. glabrata*, *C. parapsilosis*, and *C. lusitanie* (F) strains isolated from COVID-19 patients on polystyrene. (•) Without treatment with proteinase K, (•) With treatment with proteinase K (1 mg/mL).
