# Supplementary material for: Typing of Candida spp. from Colonized COVID-19 Patients Reveal Virulent Genetic Backgrounds and Clonal Dispersion
Source: Pathogens. 2023 Sep 29;12(10):1206. doi: 10.3390/pathogens12101206 (PMC10610241; doi:10.3390/pathogens12101206)
Supplement: Supplementary file 1 [file pathogens-12-01206-s001.zip › pathogens-2568561-supplementary.pdf]

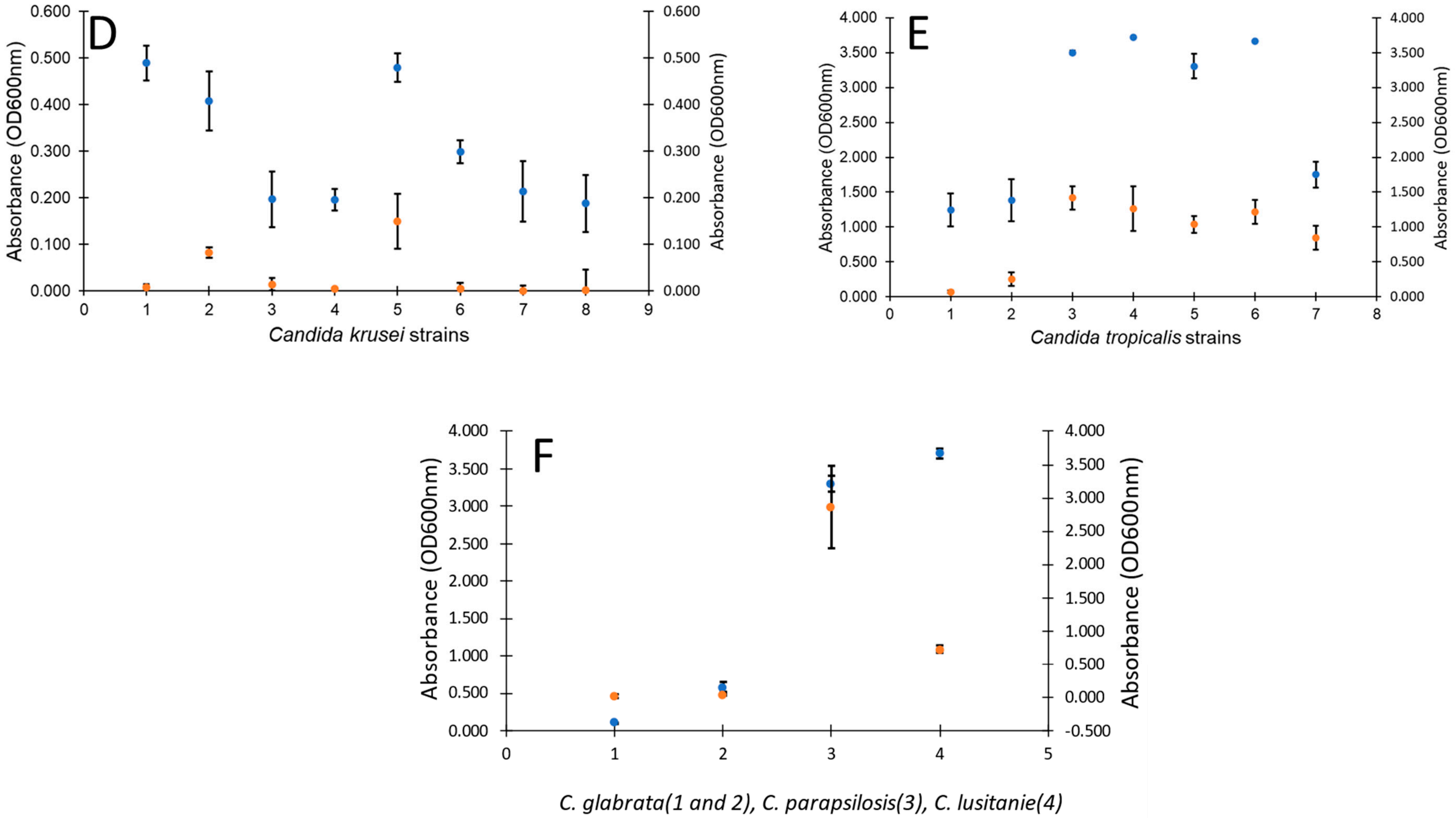

Supplementary material Figure S1. Adherent phenotype of the population of *C. krusei* (**D**), *C. tropicalis* (**E**), *C. glabrata, C. parapsilosis*, and *C. lusitanie* (**F**) strains isolated from COVID-19 patients on polystyrene. (•) Without treatment with proteinase K, (•) With treatment with proteinase K (1 mg/mL).
